# Positron emission tomography and single photon emission computed tomography imaging of tertiary lymphoid structures during the development of lupus nephritis

**DOI:** 10.1177/20587384211033683

**Published:** 2021-08-03

**Authors:** Esmaeil S Dorraji, Ana Oteiza, Samuel Kuttner, Montserrat Martin-Armas, Premasany Kanapathippillai, Sara Garbarino, Gustav Kalda, Mara Scussolini, Michele Piana, Kristin A Fenton

**Affiliations:** 1 RNA and Molecular Pathology Research Group, Institute of Medical Biology, Faculty of Health Sciences, 8016 UiT The Arctic University of Norway, Tromsø, Norway; 2 Nuclear Medicine and Radiation Biology Research Group, Department of Clinical Medicine, Faculty of Health Science, 8016 UiT The Arctic University of Norway, Tromsø, Norway; 3 Centre for Medical Image Computing, Department of Computer Science, University College London, London, UK; 4 Dipartimento di Matematica, 9302 Universita di Genova, Genova, Italy; 5 Dipartimento di Matematica, 9302 Universita di Genova, and CNR-SPIN, Genova, Italy

**Keywords:** systemic lupus erythematosus, lupus nephritis, tertiary lymphoid structures, positron emission tomography, single photon emission computed tomography, 18-F-fluoro-2-deoxy-D-glucose, 99mTC-Nanocoll

## Abstract

Lymphoid neogenesis occurs in tissues targeted by chronic inflammatory processes, such as infection and autoimmunity. In systemic lupus erythematosus (SLE), such structures develop within the kidneys of lupus-prone mice ((NZBXNZW)F1) and are observed in kidney biopsies taken from SLE patients with lupus nephritis (LN). The purpose of this prospective longitudinal animal study was to detect early kidney changes and tertiary lymphoid structures (TLS) using in vivo imaging. Positron emission tomography (PET) by tail vein injection of 18-F-fluoro-2-deoxy-D-glucose (^18^F-FDG)(PET/FDG) combined with computed tomography (CT) for anatomical localization and single photon emission computed tomography (SPECT) by intraperitoneal injection of ^99m^TC labeled Albumin Nanocoll (^99m^TC-Nanocoll) were performed on different disease stages of NZB/W mice (*n* = 40) and on aged matched control mice (BALB/c) (*n* = 20). By using one-way ANOVA analyses, we compared two different compartmental models for the quantitative measure of ^18^F-FDG uptake within the kidneys. Using a new five-compartment model, we observed that glomerular filtration of ^18F^FDG in lupus-prone mice decreased significantly by disease progression measured by anti-dsDNA Ab production and before onset of proteinuria. We could not visualize TLS within the kidneys, but we were able to visualize pancreatic TLS using ^99m^TC Nanocoll SPECT. Based on our findings, we conclude that the five-compartment model can be used to measure changes of FDG uptake within the kidney. However, new optimal PET/SPECT tracer administration sites together with more specific tracers in combination with magnetic resonance imaging (MRI) may make it possible to detect formation of TLS and LN before clinical manifestations.

## Introduction

Systemic lupus erythematosus is a systemic autoimmune disease characterized by the production of autoantibodies to nuclear self-antigens.^1,2^ In Lupus nephritis (LN) the formation of immune complexes and their consequent deposition within the kidney causes enhanced inflammation, which leads to disruption of kidney integrity, and development of significant clinical proteinuria.^3–7^ The resulting proteinuria is often discovered too late to treat and save the kidneys. This can cause acute inflammatory lesions and chronic damage to the kidney and can cause the patient to require lifetime treatment, dialysis, or transplantation.^4,5,8,9^ To evaluate the progression of LN, a renal biopsy is the gold standard, where a repeated biopsy often is required.^10,11^ Methods that can detect the early clinically silent events of LN are therefore highly needed.

Positron emission tomography is an in vivo imaging method commonly used in oncological applications, CNS, and cardiovascular imaging. One of the common tracers used in PET is the 18-F-fluoro-2-deoxy-D-glucose (^18^F-FDG). This tracer is taken up by cells that are highly glycolytic metabolically active. In addition to the neoplastic cells, immune cells, like macrophages and granulocytes, increase their expression of glucose transporters and glycolytic enzymes during phagocytosis.^12,13^ This makes the use of metabolic PET an interesting method in detecting activated immune cells in vivo. *Single photon emission computed tomography* (SPECT) is another in vivo imaging method with which ^99m^TC Albumin Nanocolloid has been used widely for the detection of the first drainage sentinel lymph nodes in cancer patients.^14,15^ The uptake is facilitated through phagocytosis by macrophages and granulocytes through phagocytosis specific receptors like the mannose receptor.^16,17^

Lack of a non-invasive diagnostic test, which can diagnose LN at an early stage, is quite evident. In vivo molecular imagings such as PET and SPECT have been used widely to investigate normal and abnormal biological processes in different organs, but little work has been done for the kidney.^18–20^ The most used PET tracer is ^18^F-FDG, and different mathematical models of ^18^F-FDG have been developed to investigate the uptake in different organs.^21–23^ However, it has been difficult to design a model to evaluate renal FDG-PET data with regards to the complex structure of the kidney and the high excretion of FDG through the kidney.^18,24,25^

The inflammatory aggregates observed during the progression of murine and human LN are organized into well-defined tertiary lymphoid structures (TLS).^26–28^ TLS form in tissues that are targeted by chronic inflammatory processes, such as infection and autoimmunity.^29,30^ We postulate that local events in the kidney, prior to development of proteinuria, are the early but causal process in LN. We hypothesize that TLS formation can be used to detect a silent mesangial nephritis without renal clinical symptoms. Since TLS share similarities with lymph nodes regarding function and structure,^31^ the aim of this study was to use the of ^18^F-FDG in PET and ^99m^TC Albumin Nanocolloid in SPECT to investigate kidney functionality during SLE disease progression and facilitate the detection of the formation of TLS within the kidney of lupus-prone NZB/W mice, a model of SLE in humans.

## Materials and methods

### Calculation of sample size

This is a prospective longitudinal animal study. The amount of the animals was calculated based on the “resource equation” method using the equation: E = Total number of animals − Total number of groups. E is the degree of freedom of analysis of variance (ANOVA), and the value of E should lie between 10 and 20 (ref). The minimum number of animals in this study was calculated to be three in each group and maximum four when using six groups of animals.

### Animals and grouping

20 BALB/c and 40 (NZBxNZW)F1(NZB/W) mice were purchased from Harlan (Harlan Sprague Dawley Inc, Indiana, USA). Forty-three of the mice were included for PET imaging, 19 mice for SPECT imaging and 16 mice for organ distribution. The Animal Welfare Board, at the UiT–The Arctic University of Norway and the regional ethical committee in Northern Norway approved all procedures (Reference number 6776).

The mice were divided into groups based on age, anti-dsDNA positivity and proteinuria. The groups consisted of 7 weeks old BALB/c (young BALB/c) (*n* = 3); 14–37 weeks old BALB/c (old BALB/c) (*n* = 11); 7 weeks old anti-dsDNA Ab negative NZBW (young Ab neg) (*n* = 4); 11–33 weeks old anti-dsDNA Ab negative NZBW (old Ab neg) (*n* = 20); anti-dsDNA Ab positive (being Ab positive for from 1 up to 6 weeks) (Ab+) (*n* = 12); and anti-dsDNA Ab positive (being Ab positive for from 7 up to 9 weeks) (Ab++) (*n* = 11). None of the mice included in PET or SPECT were proteinuric.

### Anti-dsDNA Antibody enzyme-linked immunosorbent assay and measurement of proteinuria

The level of anti-dsDNA antibody in lupus-prone mice serum was measured as described previously.^32^ Blood samples were taken every week until positive ELISA test, and thereafter, every second week until proteinuria was detected in urine. The protein in the urine was determined by urine dipstick (Bayer Diagnostics, Bridgend, United Kingdom): 0–1+ (<1 g/L) was regarded as physiological proteinuria; 2+ (≥1–3 g/L) and 3+ (≥3–20 g/L) and 4+ (≥20 g/L). Proteinuria was defined as 4+.

### Animal preparation

Mice subjected to ^18^F-FDG PET/CT imaging were fasted 3 h before acquisition, weighted, and anesthetized. Mice were anesthetized with isoflurane (induction 4% and maintenance 2% in oxygen). To prevent hypothermia, the temperature of the bed was set to 35°C during imaging procedure. For mice undergoing PET/CT imaging, a catheter with 30-gauge needle was placed into the mice lateral tail vein to allow tracer injection.

### Imaging

PET, SPECT, and CT imaging were performed by using the Triumph^TM^ LabPET-8^TM^, 4-detector configuration X-SPECT® small animal PET/SPECT/CT scanner (Trifoil Imaging, Northridge Tri-Modality Imaging, Inc., Chatsworth, CA).

### ^18^F-FDG PET/CT imaging

10.57 ± 1.79 MBq ^18^F-FDG was diluted in 100 μl in sterile saline (*n* = 103) and injected through the lateral vein catheter using an automated infusion pump (0.2 ml/min). A 60-minute list mode PET acquisition was started simultaneously with the start of the ^18^F-FDG injection. After PET acquisition, two CT scans were acquired at 80 kVp, 2 × 2 binning, 512 projections, and 1.3 × magnification. The first was performed without contrast agent, and the second with 125 µl Iomeron350 (Bracco Imaging Scandinavia AB, Sweden) injected through the lateral vein catheter using an automated infusion pump over a 60 s period. The second CT scan started 2 min post-contrast agent injection.

The acquired list mode PET data were histogrammed into 24 × 5 s, 9 × 20 s, and 11 × 5 min, in total 44 time frames, and reconstructed using 3-dimensional maximum likelihood expectation maximization (MLEM) algorithm with 50 iterations. PET images were corrected for varying detector element efficiency, radioactive decay, random coincidences, dead time losses, attenuation, and scatter, before a dose calibration factor was applied, to convert the output images into the unit of MBq/cm^3^. CT raw data were reconstructed using filtered back projection algorithm.

### ^99m^TC-Nanocoll SPECT/CT imaging

Mice were anesthetized as described above and injected intraperitoneally (i.p.) with ^99m^TC-Nanocoll (mean dose activity 56.37 MBq ± 9.1 MBq) diluted in sterile saline (100 μl maximum injection volume). Dynamic SPECT scans (60 min, 4 × 15 min time frames) started 12 ± 9 min post tracer injection. Acquisition was performed with 90° rotation, to cover one revolution with the four SPECT detectors, 16 × 4 projections, five multipinhole collimator (N5F75A10), and 45 mm radius of rotation. The images were reconstructed using ordered subset expectation maximization algorithm (eight subsets and five iterations). A CT scan was performed after SPECT acquisition, with identical acquisition parameters as for PET imaging, described above.

### Data analysis of in vivo imaging

#### Kinetic analysis of PET data

Images were analyzed using PMOD (PMOD Technologies Ltd., Zürich). Volumes of interests representing whole kidney, cortex, and renal hilum were delineated in the CT images, and VOIs representing bladder were drawn in the PET images. These VOIs were outlined semi-automatically (60% isocontour of the maximal voxel value). ^18^F-FDG blood concentrations were obtained from the vena cava VOI (1 mm^3^). This VOI was selected by finding the voxel with highest activity in the first 5–10 s of the PET scan (first or second time frame). Volumes of interest data were converted to standardized uptake value (SUV) g∗min/ml) by normalizing to injection dose per animal weight. The area-under-curve (AUC) was calculated for renal hilum, cortex, and kidney tissue regions based on the measured time activity curves from each segmented region in the dynamic PET data.

#### Three-compartment model

**Figure 1.**
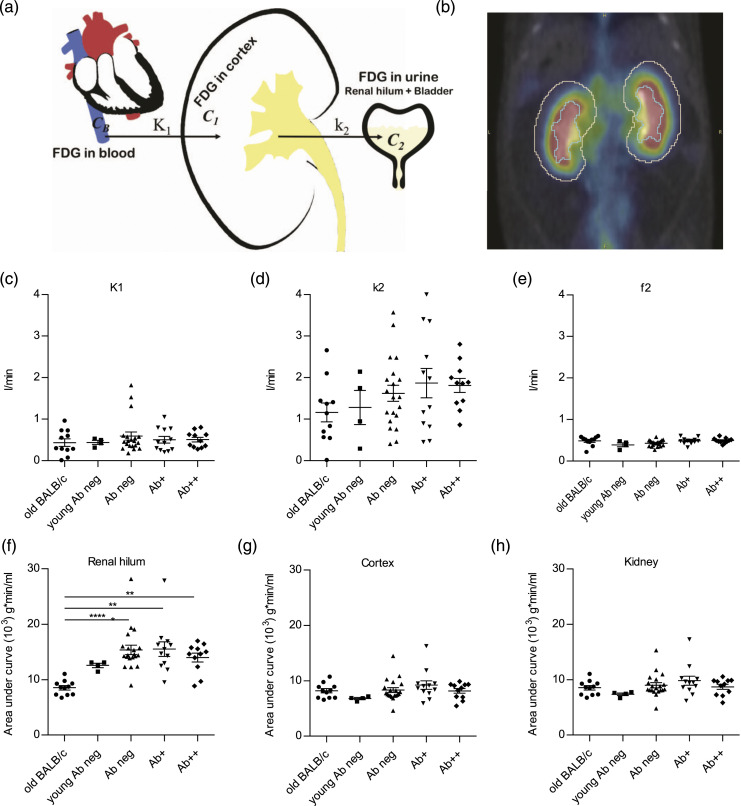
Renal hilum SUV increased in anti-dsDNA Ab+ mice. (a) Illustrates the three-compartment model. *C*_*B*_, *C*_1_, and *C*_2_ represent tracer in the blood (input function), activity concentration in the parenchyma, and urine, respectively. (b) Cortex and renal hilum were delineated based on CT. There was no difference in *K*_1_ (c) and *f*_2_ (e) between the groups, but *k*_2_ (d) increased during SLE progression. Area-under-curve of renal hilum (f) increased during SLE progression, while AUCs of cortex (g) and whole kidney (h) were steady. (c)–(h) One-way ANOVA with post-hoc analysis (Dunn’s multiple comparisons test). *p < 0.05, **p < 0.01, ***p < 0.001, ****p < 0.0001.

A three-compartment model, based on work done by Qiao et al.,^18^ was implemented in an in-house developed MATLAB script (Mathworks, USA) (Figure 1a). The model equation is given by *C*_*T*_(*t*) = *K*_1_*e*^−*k*2*t*^ ⊗ *C*_*B*_(*t*) + *f*_1_*C*_*B*_(*t*) + *f*_2_*C*_renal hilum_, where *C*_*B*_ is the input function, approximated by the image derived time activity curve (TAC) from the vena cava region. *C*_1_ and *C*_2_, corresponding to the activity concentration in parenchyma and urine, respectively, were estimated from kidney cortex and urine in renal hilum and bladder TACs, respectively. *C*_*T*_ is the concentration of FDG measured in the kidneys from PET. The model was fitted to the data using a nonlinear least-squares solver (lsqcurvefit). The resulting model parameters *K*_1_ and *k*_2_, the fractional calibration factors *f*_1_ and *f*_2_, and the coefficient of determination, *r*^2^, were extracted and compared between different age and disease stage groups of animals.

#### Five-compartment model

**Figure 2.**
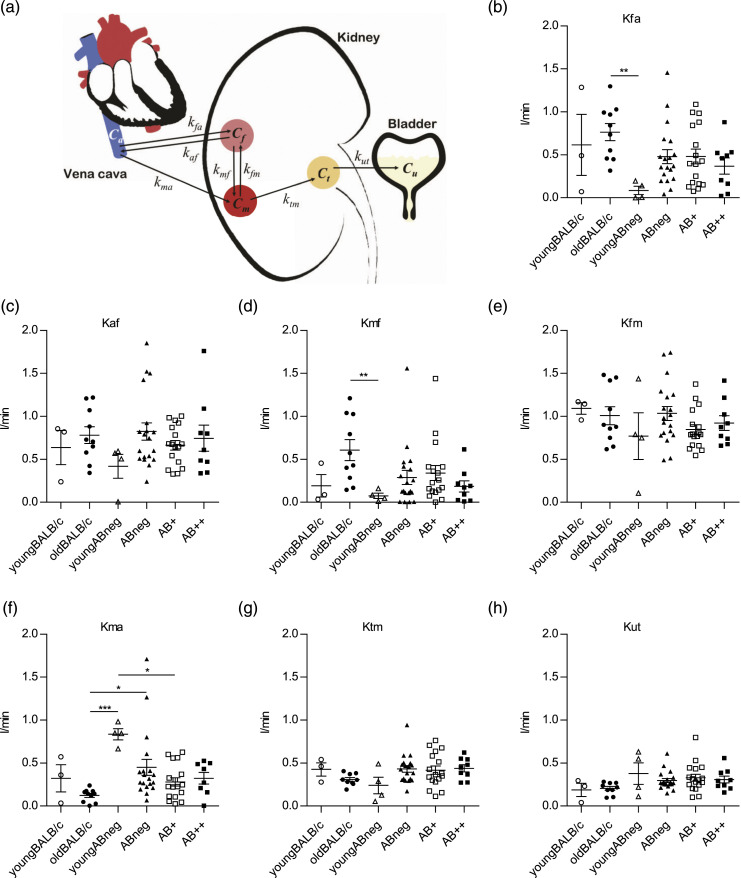
Filtration of ^18^F-FDG by glomeruli was lower in lupus-prone mice producing anti-dsDNA Ab. (a) Illustrates the five-compartment model, where *C*_*a*_, *C*_*f*_, *C*_*t*_, *C*_*u*_, and *C*_*m*_ p represent tracer in blood, extravascular, tubule, urine, and metabolized ^18^F-FDG, respectively. The *kfa* (b), *k*_*af*_ (c), and *kmf* (d) were increased in mice with anti-dsDNA Ab production, but *k*_*ma*_ (f) decreased. *K*_*fm*_ (e)*, k*_*tm*_ (g), and *k*_*ut*_ (h) were steady during SLE progression. (b)–(h) One-way ANOVA with post-hoc analysis (Dunn’s multiple comparisons test). *p < 0.05, **p < 0.01, ***p < 0.001.

The five-compartment model (Figure 2a), described by Garbarino et al.,^33^ consists of *C*_*m*_: a compartment involving the ^18^F-FDG-6-phosphate, the FDG in cells, and the pre-urine pool accounting for tracer filtered in the pre-urine and entering the proximal tubule. This compartment contains the majority of tracer in the kidneys, which is defined as a metabolized compartment to indicate the fact that the FDG can also be in the ^18^F-FDG-6-phosphate form in this state. *C*_*a*_: vena cava compartment, which includes tracer in blood flow that later enters to the kidney. *C*_*f*_: an extravascular compartment in the kidney representing the free tracer outside cells, where they exchange with vena cava and metabolized compartments. *C*_*t*_: the tubular compartment, in which tracer concentration can vary because of water reabsorption. *C*_*u*_: accounting for the ultimate tracer excretion, which has been measured in urine in the bladder.

The exchange rates are denoted as *k*_*XY*_, where _*X*_ indicates the compartment of destination and _*Y*_ the compartment of origin. Simple balance of tracer leads to the following set of equations

$$C_{f}=-\left(k_{\mathrm{af}}+k_{\mathrm{mf}}\right)C_{f}+k_{\mathrm{fm}}C_{m}+k_{\mathrm{fa}}C_{a}$$

$$C_{m}=k_{\mathrm{mf}}C_{f}-\left(k_{\mathrm{fm}}+k_{\mathrm{tm}}\right)C_{m}+k_{\mathrm{ma}}C_{a}\;$$

$$C_{t}=-k_{\mathrm{ut}}C_{t}+k_{\mathrm{tm}}C_{m}$$

The total concentration of tracer in the kidneys, C_K_, can be written as (Kimura et al.^34^)

$$C_{K}=\eta_{a}C_{a}+\left(1-\eta_{a}-\eta_{t}\right)\left(C_{f}+C_{m}\right)+C_{t}\eta_{t}\;$$

where η_*t*_ and η_*a*_ are the fractions of kidney volumes *V*_*K*_ occupied, respectively, by the tubular compartment and the blood. The model was implemented in an in-house developed MATLAB script. Groups of mice consisted of youngBALB/c (*n* = 3), oldBALB/c (*n* = 10), young Ab neg (*n* = 4), Ab neg (*n* = 19), Ab+ (*n* = 17), and Ab++ (*n* = 9).

#### SPECT/CT data analysis

The images obtained from ^99m^TC-Nanocoll dynamic SPECT were visually evaluated by three nuclear medicine *specialists* to identify the accumulation of the colloid in the different organs in different groups: BALB/c (*n* = 3), anti-dsDNA Ab− (*n* = 8), and anti-dsDNA Ab++ (*n* = 10). Volumes of interests were drawn automatically in the entire mouse in the 4^th^ SPECT time frame. This was achieved by using 3D automatic segmentation at 25% threshold of the maximum signal inside the mouse body. All VOIs were quantified by mean pixel intensity, and those with accumulation of ^99m^TC-Nanocoll over time were selected for further analysis (Supplementary Figure S1).

### Ex vivo organ biodistribution

The anesthetized animals were euthanized by cervical dislocation 1 h after ^18^F-FDG injection. Internal organs were collected, rinsed (2× deionized water), weighted, and measured for radioactivity in an automatic gamma counter (2480 Wizard, PerkinElmer, MA, USA). Organ SUVs were calculated as described above.

### Immunohistochemistry

Hematoxylin and eosin (H&E) staining, *Alcian blue*/*periodic acid–Schiff* (AB/PAS) staining, and detection of CD3 positive T cells (Dako, 3 μg/ml), B220 positive B cells (R&D systems, 0.83 μg/ml), and F4/80 positive macrophages (Biolegend, 1 μg/ml) in 4 μm paraffin embedded kidney sections were performed as described previously.^28,35^ Envision+ system-HRP/DAB anti-rabbit detection kit (K4011, Dako) was used to detect CD3, and Polink-2 Plus HRP anti-rat DAB detection kit (D46-15, Golden Bridge International) was used to detect B220 and F4/80.

### Autoradiography

Mice were anesthetized with isoflurane and buprenorphine injected subcutaneously (Temgesic, 0.1 mg/kg body weight). Following ^99m^TC-Nanocoll SPECT/CT imaging, *ex vivo* autoradiography of pancreas was performed. A blood sample was collected by cardiac puncture, and mice were euthanized by blood loss under anesthesia. Subsequently, pancreas was removed, embedded in Tissue-Tek® O.C.T. (Sakura-Finetek, USA), and frozen on dry ice. Sections (−20°C, 20 μm thick) were prepared by a cryostat and thaw-mounted on Superfrost^®^ Plus glass slides (Carl Roth GmbH & Co. KG, Karlsruhe, Germany). Sections were thawed and dried for 30 minutes at 22°C. Slides were exposed to a phosphor storage screen inside a cassette overnight at 21°C. Images were developed using a Cyclone Plus storage phosphor system (Perkin Elmer, MA, USA), and the results were evaluated using Image J software (National Institutes of Health, MD, USA). A calibration factor was calculated after counting the radioactivity in the blood samples and normalizing to injection dose per animal weight. Image pixel values were converted to SUV.

### Reagents and antibodies

^18^F-FDG was provided by the University hospital of Northern Norway (UNN)(MAP Medical Technologies, Finland). ^99m^TC and Nanocoll were purchased from GE Healthcare (USA). CD3 rabbit-anti-mouse (AD452) was purchased from Dako (Denmark)), B220 rat-anti-mouse (MAB1217) from R&D Systems (USA), and F4/80 rat-anti-mouse (122603) was obtained from Biolegend (USA).

### Statistical analysis

One-way ANOVA with post-hoc analysis (Dunn’s multiple comparisons test) was performed for PET and SPECT, and unpaired t test was performed on ex vivo organ biodistribution data. A *p*-value of ≤0.05 was considered statistically significant, and data are presented as mean ± SEM. Statistical analysis was performed with GraphPad Prism (GraphPad Software, CA, USA).

## Results

### Decreased glomerular filtration was detected by ^18^F-FDG PET during early SLE progression

To investigate kidney functionality during SLE disease progression, dynamic PET/CT was performed on different groups of lupus-prone mice (NZBW F1) and control BALB/c mice. The 3CM, using vena cava, kidney, and urine, was used to analyze the data (Figure 1a). Two VOIs, cortex and renal hilum, were defined in kidneys based on the CT (Figure 1b). The *K*_1_ and *f*_2_ generated with MATLAB 3CM were the same in BALB/c and NZBW at the different stages of disease (Figure 1c and e). The *k*_2_ increased synchronously with disease progression in NZBW mice, and the highest levels were observed in the Ab+ and Ab++ groups. The AUC of the renal hilum was higher in Ab neg, Ab+, and Ab++ compared to young Ab neg and significantly higher compared to old BALB/c (Figure 1f). There were no significant differences of AUC for the cortex and the whole kidney (Figure 1g and h, respectively).

To determine the best possible compartment model to evaluate the kidney functionality during SLE disease progression, data generated through dynamic PET were analyzed by the 5CM (Figure 2a). The *k*_*fa*_, *k*_*af*_, and *k*_*fm*_ were higher in Ab neg, Ab+, and Ab++ groups compared to young Ab neg group (Figure 2b–d). A significantly lower level of *k*_*ma*_ was observed in Ab+ compared to young Ab neg, and it was notably lower in Ab neg compared to young Ab neg (Figure 2f). The *k*_*mf*_*, k*_*tm*_, and *k*_*ut*_ were relatively the same in Ab neg, Ab+, Ab++, and young Ab neg groups (Figure 2g and h). The mean *k* values with SEM for 1 BALB/c and 3 different NZB/W mice are shown in Supplementary Figure S2.

### ^18^F-FDG accumulate highly in lymph nodes of anti-dsDNA Ab+ Mice

**Figure 3.**
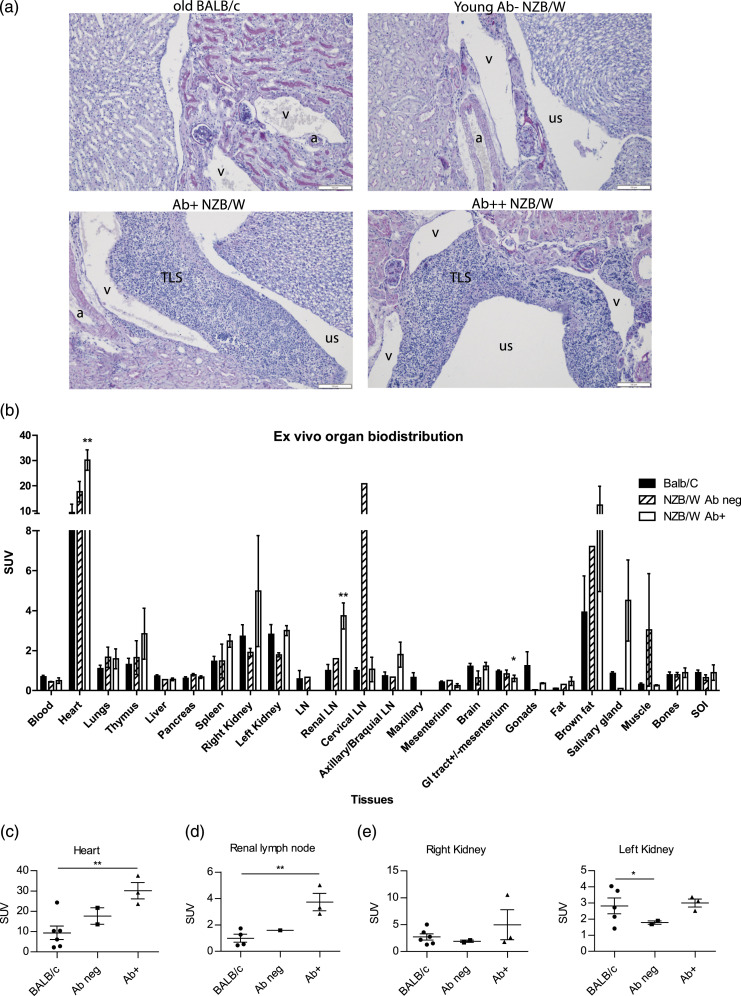
Histological pictures and ex vivo organ biodistribution. (a) Representative *Alcian blue*/*periodic acid–Schiff* (AB/PAS) stained kidney sections from old BALB/c, Ab- NZB/W, Ab+ NZB/W, and Ab++ NZB/W mice. (b) ^18^F-FDG accumulation measured by SUV (g/ml) in tissue after 24 h. ^18^F-FDG accumulated higher in heart (c) and renal lymph node (d). ^18^F-FDG was higher in right (e) and left (f) kidneys, axillary/brachial lymph node, spleen, thymus, brain, and salivary gland (b) of anti-dsDNA Ab+ mice compared to Ab neg mice and was lower for GI track (b). Unpaired *t* test. *<0.05, **<0.01. a, artery; us, urinary space; v, vein. Scale bar = 100 µm.

Ex vivo organ biodistribution studies after dynamic PET were performed on BALB/c (*n* = 6), Ab neg (*n* = 2), and Ab+ (*n* = 3) mice to examine ^18^F-FDG accumulation in different organs of different groups of mice (Figure 3a). We observed a notable higher ^18^F-FDG accumulation in the heart (Figure 3b) renal lymph nodes (Figure 3c), axillary/brachial lymph nodes, right and left kidneys (Figure 3d and e, respectively), spleen, thymus, and salivary gland in Ab+ compared to Ab neg mice (Figure 3a). The SUV value for gastrointestinal tract was significantly lower in Ab+ NZB/W compared to BALB/c and moderately lower compared to Ab neg mice (Figure 3a). Standardized uptake value measured in gonad, brown fat, fat, liver, lung, muscle, bone, pancreas, and SOI was relatively the same between the groups (Figure 3a).

### ^99m^TC-Nanocoll SPECT can visualize organ-specific TLS

**Figure 4.**
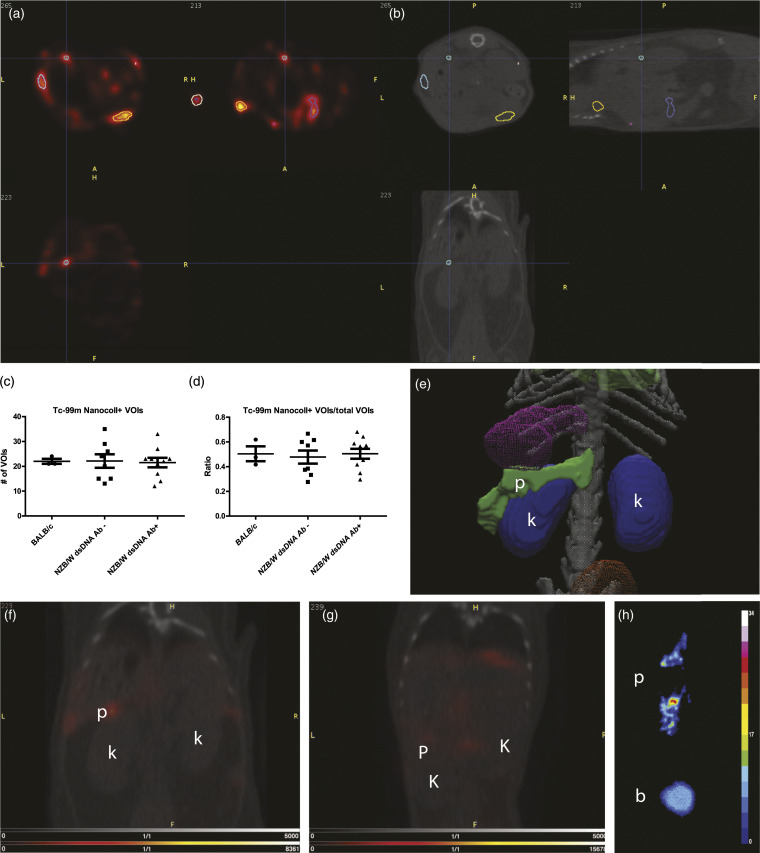
SPECT imaging of pancreatic TLS. (a) SPECT and (b) CT transversal, coronal, and sagittal frames of an example (dsDNA Ab+) showing multiple ROIs generated in Pmod 3.7. A threshold of 75% of the maximum activity was defined for the delineation in all mice. (c) Total number of VOIs accumulating ^99m^TC-Nanocoll over time in BALB/c, dsDNA Ab- NZB/W, and dsDNA Ab+ NZB/W groups. (d) Relation between total number of VOIs drawn and the number of VOIs that accumulated ^99m^TC-Nanocoll with time. No differences among the groups could be observed. (e) Atlas representation of kidneys and pancreas in the mouse. (f) Representing a fusion SPECT/CT image of the NZB/W dsDNA Ab+ mouse with a hot spot in the pancreatic area, which could only be observed in NZB/W dsDNA Ab+ mice (*n* = 6) not in the (g) BALB/c or NZB/W dsDNA Ab neg mice (data not shown). (h) Autoradiography of the ^99m^TC Nanocoll in dsDNA Ab+ mice, which shows a clear tracer accumulation in the pancreas in comparison to blood. (c)–(d) One-way ANOVA with post-hoc analysis (Dunn’s multiple comparisons test). p, pancreas; k, kidney; b, blood.

The ^99m^TC-Nanocoll was used to investigate the presence of TLS in organs of lupus-prone mice at different stages of disease progression. We observed no differences among the total number of VOIs automatically drawn by the software in the different groups, and no differences in the number of VOIs positively accumulating Nanocoll with time among the groups (Figure 4a–d). Through all the positive VOIs within the different groups, only the VOI which was drawn in the pancreatic area varied between groups. The pancreatic VOI was only observed in the dsDNA Ab+ group (6 out of 11 mice), which was selected due to its notable high signal intensity (Figure 4f). We did not observe the same observation in the anti-dsDNA Ab neg and BALB/c groups (Figure 4g).

**Figure 5.**
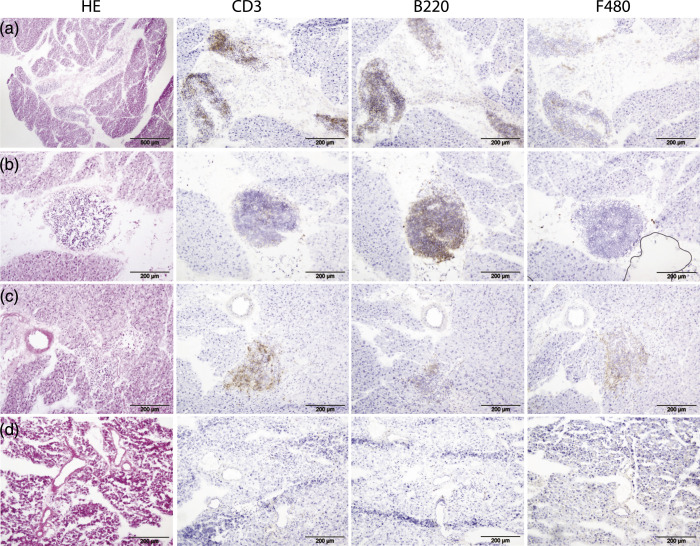
HE staining and immunostaining of TLS in pancreatic tissue in lupus-prone (a)–(c) and BALB/c mice (d). TLS was positively stained for T cells (CD3), B cells (B220), and macrophages (F480). Some TLS was detected near pancreatic islets (black arrows, c). (d) TLS was not detected in pancreatic sections from BALB/c mice. a, artery; d, ducts.

The ex vivo autoradiography following dynamic SPECT was performed to confirm the accumulation of ^99m^TC-Nanocoll in pancreas of anti-dsDNA Ab+ (*n* = 4) and BALB/c (*n* = 2) groups. We detected the accumulation of colloid in pancreas of dsDNA Ab+, but not in BALB/c (Figure 4h). We performed HE staining and IHC detecting T cells, B cells, and macrophages on pancreatic sections from NZB/W and BALB/c mice (Figure 5). In the mice with positive SPECT signals, we found TLS within the pancreas (Figure 5a–c). The TLS were detected within stromal areas (Figure 5a and b), but also within the pancreatic tissue close to the larger arteries and veins (Figure 5c). The BALB/c mice did not show any infiltration of aggregated immune cells (Figure 5d).

## Discussion

In this study, we have performed dynamic PET-CT, SPECT-CT, ex vivo organ biodistribution, and autoradiography on lupus-prone mice at different stages of the disease to develop non-invasive molecular imaging techniques to visualize TLS, measure changes in FDG uptake, and diagnose LN at an early stage. The AUC-SUV for renal hilum and SUV value for k2 (urinary clearance of FDG) increased during LN progression. Hao et al.^18^ have reported a decline in the time of FDG clearance in anti-GBM nephritic mice by disease progression, but they did not evaluate the level of FDG clearance regardless of the time. In the 3CM model, the metabolized FDG was neglected based on the small amount compared to FDG in urine or free FDG.^18^ In a healthy kidney, it might be correct, but in autoimmune patients with progressive nephritis owing to a large population of cells with high metabolic needs such as activated macrophage, B cell and T cell in the kidney,^36,37^ the metabolized FDG cannot be ignored. The 3CM model was developed based on human kidneys and regard to the fundamental differences between human and mouse kidney; this model might not be the best choice for evaluating preclinical FDG-PET data.

The majority of previous renal system compartment models have defined two functional compartments, which are anatomically embedded.^33^ However, 5CM in this study introduces the tubular (functional and anatomical) and bladder (anatomical) compartments.^33^ Garbarino et al.^33^ designed the model based on an increase of *k*_*ut*_ compared to *k*_*tm*_ by the factor of 10 due to water reabsorption in the tubule. In the 5CM in this study, the volume of water and kidney is considered as a fixed value. Thus, *k*_*ut*_ will not increase compared to *k*_*tm*_ due to water reabsorption. Since the tracer will not be reabsorbed in the tubule and we have a fixed value for the volume of water and kidney, *k*_*tm*_ and *k*_*ut*_ should represent relatively the same level as we observed in our data. We consider it as a validation for the persistent accuracy of data generated by the 5CM model.

Through extensive glomerular damage in LN, the glomerular filtration is disturbed by disease progression.^37,38^ In the 5CM, the *k*_*ma*_ is described as glomerular filtration. The notable decline that we observed in glomerular filtration of FDG in older Ab neg mice compared to young Ab neg mice can be considered as an early diagnostic sign of nephritis, which decreased significantly by disease progression, without clinical symptoms. Metabolized ^18^F-FDG (^18^F-FDG-6-phosphate) can stay in cells (*C*_*m*_) until the ^18^F decays and converts to ^18^O, and ^18^O-glucose-6-phosphate can be metabolized as ordinary glucose or it can be transferred to the interstitial space (*k*_*fm*_). We did not observe significant changes in *k*_*mf*_ and *k*_*fm*_ (*C*_*f*_ ⇌*C*_*m*_) during early LN progression. A weakness of this model is that it does not consider the lymphatic pharmacokinetics within the kidneys.

Systemic lupus erythematosus is an autoimmune disorder, which immune cells in secondary lymphoid organs (SLO), lymph nodes, and spleen are in an active stage. Ex vivo organ biodistribution showed higher FDG accumulation in organs with higher immune cell compositions such as lymph nodes, spleen, and thymus compared to other organs like brain, liver, and lungs. It has been reported that LN patients develop TLS in the kidney,^39,40^ and based on our finding, anti-dsDNA Ab positive lupus-prone mice develop TLS within the kidney, and when they become proteinuric, a large and organized TLS with germinal center-like structures develop within the kidney.^26,28^ In this study, we observed higher ex vivo FDG accumulation in the kidney of anti-dsDNA Ab positive groups compared to Ab neg mice, which are in accordance with the presence of large population of metabolic active or activated immune cells. However, the tracer was injected intravenously (i.v.), and it is unclear if this route of administration will reach the developing TLS within the kidney or other organs. Based on our previously published research, the TLS develop in near proximity to the biggest veins and arteries and contain lymph vessels and micro capillaries.^26,28^ The larger renal drainage lymphatics in mice can be found in the same hilar area.^28,41^ However, it is still unknown if the blood flow in TLS is connected to the larger vessels. Very little is known about the lymphatic flow within TLS.^42^ Based on our results, it seems like the tracer did not reach the TLS. To detect formation of TLS within the kidney, more knowledge about the lymphatics within the kidney and possible injection sites to reach them are needed.

TLS resemble SLO regarding structure, cell composition, and function.^31^ Labeled radiotracers up to about 100 nm can enter the lymphatic capillaries and be transported to lymph nodes.^43^ More than 95% of the nanocolloidal albumin particles are up to 80 nm, which make them an excellent candidate to study lymph nodes with high uptake rate.^44,45^ Our data showed accumulation of ^99m^TC Nanocoll in the lymph nodes and spleen after 1 h. Several studies have reported pancreatic TLS contains T and B cell zones, HEVs, plasma cells, DCs, and HEVs in different mice models.^46–49^ We demonstrated for the first time that ^99m^TC Nanocoll accumulated in the pancreas of anti-dsDNA Ab positive NZB/W mice. The TLS within the pancreas was positively immune stained for macrophages, T and B cells, especially those located in the edge of the pancreas. Immune aggregates within the pancreas have been reported in NZB and NZB/W mice.^50^ Several studies have previously reported incidences of pancreatitis in SLE patients,^51^ and some SLE patients had initial pancreatitis as an early manifestation of the disease.^52^ As for the pathogenic role of TLS and pancreatitis in murine SLE, the immune infiltrates were found around the pancreatic ducts, arteries, and veins and not close to the islets.^50^ This is not in accordance with our study where TLS was located close to islets. In the previous studies, most of animals and patients had normal glucose levels, and most of the patients responded well to anti-inflammatory treatments.^53^ Hence, the development of TLS within the pancreas of lupus-prone mice and SLE patients may reflect the systemic inflammatory disease caused by autoantibody binding in arteries and veins. We could not detect increased uptake of ^99m^TC Nanocoll in TLS within the kidneys. This could be due to the i.p. injection of the tracer or more likely due to the location of the TLS within the kidneys. Since the mice investigated had large TLS within the kidney, the i.p. injected tracer most likely did not reach the structure. This could be because of the direction of the blood and lymphatic flow within the kidney as discussed for the PET data. However, more studies on TLS formation in SLE are needed.

There are limitations in our study. The 18F FDG tracer has a limitation of use because of the relatively short half-life. This, together with the clearance of tracer by the kidney, makes it difficult to measure and visualize uptake of the tracer within structures of the kidney. In addition, the selected site of injection for both the PET and SPECT tracers may not be optimal for reaching the TLS within the kidney. In rodents, i.p. of macromolecules is typically taken up by the peritoneal lymphatics which directly drain into the thoracic duct and then empties into the venous blood supply.^54^ The renal lymphatics are bypassed by this route of administration, and the spleen that filters the blood therefore picks up these nanocolloids and delivers them to the pancreaticosplenic lymphatics, which is where we visualized TLS. Given the organization of the lymphatics with respect to the hemovascular system, the bioavailability to the kidney lymphatics is quite low with i.p. or i.v. administration. TLS have HEVs that are specialized for naive T and B cell entrance,^28^ and hence, the blood vasculature may not be an appropriate route for administration for evaluating TLS. Lymph always empties into the blood stream; it is not circulatory but rather unidirectional; and hence TLS identification in the kidney may require more careful consideration of route of administration. Our data indicate that there is a lack of lymphatic watersheds to the kidneys while the spleen lymphatics drain to the pancreatic lymphatics, explaining the tracer uptake within TLS in the pancreas. However, it is still unknown if the use of a long-lived tracer will omit the problem visualizing the uptake of tracer within TLS. The structure of the TLS developing within the kidney is relatively large compared to the whole kidney and is different in cellular density. This opens for the use of magnetic resonance imaging (MRI) in detecting TLS. Either alone or in combination with more specific tracers or appropriate injection sites.

We did not use power calculations to estimate the sample size. However, since it was not possible to assume the standard deviation and the effect size, we used the “resource equation” approach as an alternative approach to the power analysis.

## Conclusion

The new 5CM demonstrated a significant change in the uptake of FDG in kidney of non-proteinuric anti-dsDNA Ab positive mice compared to healthy control mice. We were not able to in vivo visualize TLS within the kidney of lupus-prone NZB/W mice neither by PET nor SPECT using i.v. and i.p. injection of ^18^F FDG and ^99m^TC Nanocoll, respectively. However, we observed positive uptake of ^99m^TC Nanocoll by cells within pancreatic TLS. New long-lived specific tracers in combination with MRI, along with optimal injections sites, detecting the formation of TLS, may lead to early detection of LN prior to clinical manifestations.

## Supplemental Material

sj-pdf-1-iji-10.1177_20587384211033683 – Supplemental Material for Positron emission tomography and single photon emission computed tomography imaging of tertiary lymphoid structures during the development of lupus nephritis Click here for additional data file. Supplemental Material, sj-pdf-1-iji-10.1177_20587384211033683 for Positron emission tomography and single photon emission computed tomography imaging of tertiary lymphoid structures during the development of lupus nephritis by Esmaeil S Dorraji, Ana Oteiza, Samuel Kuttner, Montserrat Martin-Armas, Premasany Kanapathippillai, Sara Garbarino, Gustav Kalda, Mara Scussolini, Michele Piana and Kristin A Fenton in International Journal of Immunopathology and Pharmacology
