# Supplementary material for: Positron emission tomography and single photon emission computed tomography imaging of tertiary lymphoid structures during the development of lupus nephritis
Source: Int J Immunopathol Pharmacol. 2021 Aug 3;35:20587384211033683. doi: 10.1177/20587384211033683 (PMC8351034; doi:10.1177/20587384211033683)
Supplement: sj-pdf-1-iji-10.1177_20587384211033683 – Supplemental Material for Positron emission tomography and single photon emission computed tomography imaging of tertiary lymphoid structures during the development of lupus nephritis [file sj-pdf-1-iji-10.1177_20587384211033683.pdf]

Supplementary Figures

A B

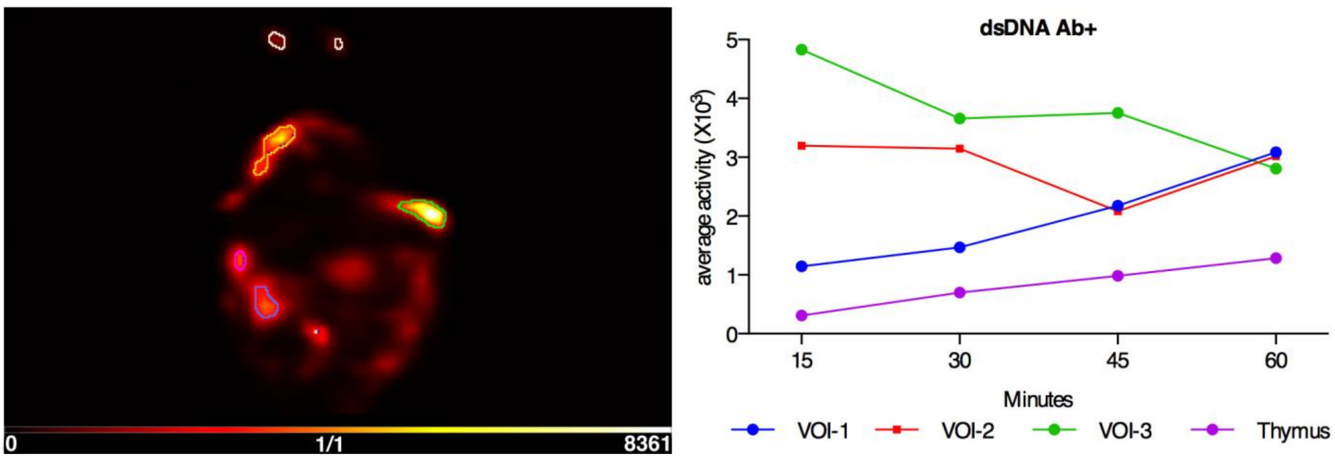

Supplementary Figure S1: Nanocoll+ VOIs selection procedure. (a) Representative SPECT image of a ^{99m}TC-Nanocoll injected mouse. VOIs were drawn based on hot searching mode with a threshold of 25%. (b) The average of the activity in the different VOIs was quantified in the different time frames. Several regions presented accumulation of ^{99m}TC-Nanocoll (VOIs 1and thymus) over time, while others showed no increase (VOI 2) or even a decrease of the average activity (VOI 3). Only VOIs with Nanocoll accumulation over time were selected for further analysis. Thymus is shown as example of linear Nanocoll accumulation.

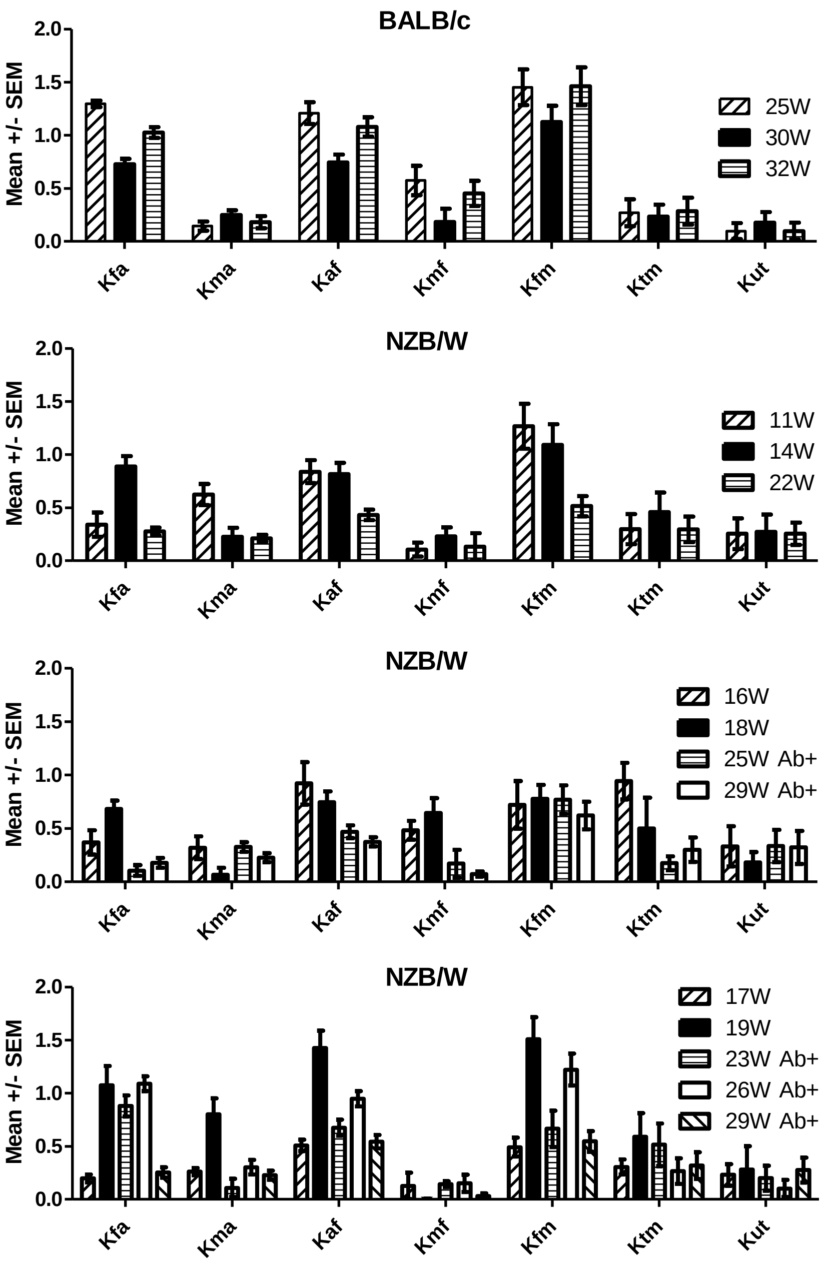

Supplementary Fig S2. Individual mice measurement of one BALB/c and three NZB/W mice scanned at 3-5 different time points. Graphs are showing mean +/- SEM of each parameter measured 20 times with Five-compartment model

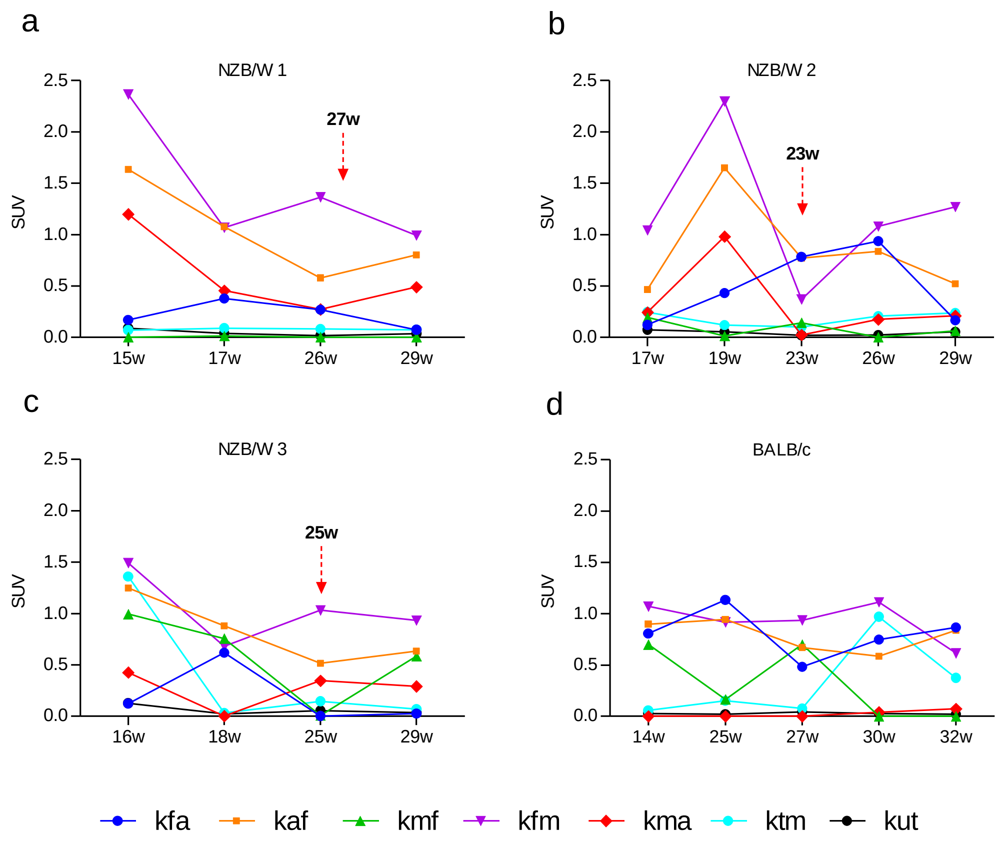

Supplementary Fig S3: k_{ma} decreases during SLE progression. Longitudinal ^{18}F-FDG PET imaging at the different age, and disease stage, on (A-C) lupus-prone mice demonstrated that different *k* values especially k_{ma} decrees by production of dsDNA Ab production. Aging did not affect *k* values in (D) BALB/c mice.
